# Improving global health governance to combat counterfeit medicines: a proposal for a UNODC-WHO-Interpol trilateral mechanism

**DOI:** 10.1186/1741-7015-11-233

**Published:** 2013-10-31

**Authors:** Tim K Mackey, Bryan A Liang

**Affiliations:** 1 Department of Anesthesiology, University of California, San Diego School of Medicine, 200 W. Arbor Drive, San Diego, CA 92103-8770, USA; 2 San Diego Center for Patient Safety, University of California San Diego School of Medicine, San Diego, USA; 3 Institute of Health Law Studies, California Western School of Law, San Diego, USA

**Keywords:** Counterfeit medicines, Global health governance, Global health policy, Drug supply chain, Falsified medicines, Fraudulent medicines, Substandard medicines

## Abstract

**Background:**

Perhaps no greater challenge exists for public health, patient safety, and shared global health security, than fake/falsified/fraudulent, poor quality unregulated drugs, also commonly known as “counterfeit medicines”, now endemic in the global drug supply chain. Counterfeit medicines are prevalent everywhere, from traditional healthcare settings to unregulated sectors, including the Internet. These dangerous medicines are expanding in both therapeutic and geographic scope, threatening patient lives, leading to antimicrobial resistance, and profiting criminal actors.

**Discussion:**

Despite clear global public health threats, surveillance for counterfeit medicines remains extremely limited, with available data pointing to an increasing global criminal trade that has yet to be addressed appropriately. Efforts by a variety of public and private sector entities, national governments, and international organizations have made inroads in combating this illicit trade, but are stymied by ineffectual governance and divergent interests. Specifically, recent efforts by the World Health Organization, the primary international public health agency, have failed to adequately incorporate the broad array of stakeholders necessary to combat the problem. This has left the task of combating counterfeit medicines to other organizations such as UN Office of Drugs and Crime and Interpol in order to fill this policy gap.

**Summary:**

To address the current failure of the international community to mobilize against the worldwide counterfeit medicines threat, we recommend the establishment of an enhanced global health governance trilateral mechanism between WHO, UNODC, and Interpol to leverage the respective strengths and resources of these organizations. This would allow these critical organizations, already engaged in the fight against counterfeit medicines, to focus on and coordinate their respective domains of transnational crime prevention, public health, and law enforcement field operations. Specifically, by forming a global partnership that focuses on combating the transnational criminal and patient safety elements of this pre-eminent global health problem, there can be progress against counterfeit drugs and their purveyors.

## Introduction

A global debate has emerged about the appropriate means to address the illicit trade in what have traditionally been known as 'counterfeit medicines.’ Ideological and economically motivated arguments over public health concerns, intellectual property rights (IPRs), and equitable access to medicines have resulted in a plethora of related but varying terms attempting to define this problem, including: 'spurious,’ 'substandard,’ 'falsified,’ 'falsely-labeled,’ 'fraudulent,’ 'unregistered,’ 'counterfeit,’ 'fake’ and the collective term 'substandard/spurious/falsely-labeled/falsified counterfeit medical products’ (SSFFC) [1]. The variety of terms utilized illustrates the numerous, complex, and fractious intersecting political, ideological, and policy issues embedded within the term 'counterfeit medicines’ [1,2], yet ongoing arguments over terminology have distracted attention from the actual global health crisis, namely, the continued widespread availability of counterfeit medicines in a variety of settings that are dangerous to public health. At present, no inclusive global health governance structure exists to mobilize health diplomacy and the multitude of stakeholder resources to combat this serious form of transnational pharmaceutical crime that affects population health [3]. This despite the fact that as early as 1988, the World Health Assembly (WHA) of the World Health Organization (WHO) had called for international action against counterfeit medicines in the interest of global medicine safety [3].

However, with ongoing breaches in the global drug supply chain and increasing detection, it appears that key global policymakers have begun to recognize the significant public health risks associated with counterfeit medicines [4]. This includes renewed activities by UN specialized agencies and other international organizations, as well as patient safety groups, law enforcement, civil society, and the private sector, among others [4-9]. Although momentum for international action against this public health hazard is building, the existing and emerging initiatives lack policy coherence, and have failed to coalesce around a unified purpose to protect patient safety and bring about the necessary international co-operation. As these problem, policy, and political streams join together, they provide the opportunity for exploration of enhanced global health governance structures to address this pre-eminent global health concern [10].

### Global scope of counterfeit medicines

Harm arises from a wide spectrum of detected dangerous counterfeit medicines across therapeutic classes, with quality, manufacturing, and/or provenance issues that make the product ineffective and/or harmful. In this discussion, we focus on the subset of 'dangerous counterfeit medicines,’ which are intentionally substandard, ineffective, or adulterated, as well as those instances where there is criminal intent to deceive regarding the authenticity or origin of the medicine. In the case of dangerous counterfeit medicines, there is a clear public health risk, given that these products can be harmful to health, there is fraud or criminal intent involved, and/or the authenticity/quality of the medicine cannot be assured. This differs from instances where medicines are unintentionally substandard and do not meet the legally required quality specifications (such as, such as an error in authorized manufacturing) [1]. In these unintentional cases, legal principles of negligence can apply, and, if reckless or egregious, possible criminal sanctions may also apply. However, for illicit activities and intentional fraud, a range of other remedial activities must also be explored to determine an appropriate regulatory and legal response.

Most importantly, dangerous counterfeit medicines place all patients at risk, from developed to developing countries, from formal and informal economy sectors, from rural clinics to tertiary care centers, and from resource-poor to high-income settings [2,11]. However, global trafficking of counterfeit medicines is difficult to quantify, largely because of the criminal element of the trade and lack of adequate surveillance [2,3]. Previous crude estimates indicated that 10% of global medicines are counterfeit, although experts acknowledge the imprecision, regional variation, and general paucity of data behind this claim [12,13]. A report from the Organisation for Economic Co-operation and Development (OECD) also highlighted the difficulty in assessing counterfeit medicines, owing to lack of data, divergent terminology, and the standard tenet that covert illegal activity is difficult to measure [14].

Available estimates have placed the global market for counterfeit medicines at between US$75 and US$200 billion, indicating that this is a multibillion dollar illicit enterprise, but also highlighting the wide range and general lack of reliable information on the topic [12,15]. Similarly, the United Nations Office of Drugs and Crime (UNODC) estimated that the market for counterfeit anti-malarial drugs was more than US$400 million in west Africa alone, a region of the world where drug regulatory systems have poor technical capacity and governance [16,17]. OECD also noted expansion and increasing diversity of medicines counterfeited, and an increasing counterfeit presence in supply chains even in strongly regulated countries [14]. Finally, WHO itself estimated that the prevalence of counterfeit medicines ranges from less than 1% in developed countries, to 10 to 30% in developing markets, and up to 50% or more from websites that conceal their physical location [11,18].

Safety dangers are further amplified by the advancement in technology and the frenetic pace of globalization, rendering international borders defenseless. Indeed, a primary mechanism for counterfeit medicine distribution and sourcing is the Internet, which is rarely subject to effective international oversight [19,20]. Instead, individuals from virtually any country with online access can search for and potentially purchase pharmaceuticals, including essential drugs, vaccines, controlled substances, life-saving medicines for serious conditions, and lifestyle products, all from the convenience of a computer or even from a mobile device [2,14,19,21-26]. Addressing online counterfeit distribution using multi-sector efforts such as the enforcement action Operation Pangea I-VI of the International Criminal Police Organization (Interpol) has resulted in millions of counterfeit pill seizures and some 40,000 websites shut down, but appears to have not stemmed the continued proliferation of illicit online pharmacies [20,27].

Publicly available data collected by the Pharmaceutical Security Institute (PSI), a not-for-profit organization of pharmaceutical industry security directors collecting and analyzing information on global pharmaceutical crime (which, in addition to counterfeit incidents, includes illegal pharmaceutical diversion and theft), also shows increasing criminal activity: an alarming 77% overall global increase in incidents of pharmaceutical crime were verified from 2005 to 2011 (1,123 to 1,986 incidents) involving 532 different pharmaceutical products [28,29]. PSI information is based on an incident-based reporting system, verified in a similar fashion to information collected by law enforcement agencies, and is sourced from PSI pharmaceutical member and non-member companies, law enforcement agencies, healthcare/regulatory agencies, and open-source media reports. To be included, a counterfeit product, using WHO definitions, must be supported by factual information validated by a team of multilingual criminal analysts from PSI [30].

However, these measurements are likely to be incomplete, and may underestimate the scope of the issue. Even beyond the challenges in assessing criminal activity, there is difficulty in assessment in regions such as Africa, which suffer from low surveillance and reporting. Further, trade in counterfeit medicines often affects more than one country, making detection and accurate reporting of origin, transit countries, and destination difficult. In addition, measurement limits such as difficulty in detection, reporting bias across regions, lack of laboratory capacity to confirm that the product is counterfeit, and lack of comprehensive data surveillance sources, mean that conclusions from such data are limited. These limitations emphasize the need for better data collection, more robust monitoring and surveillance, and a requirement for mandatory reporting at the individual country level to better inform researchers and policymakers about the scope of this global health problem.

### Limitation of current public health efforts

Although the available data describing the problem of global counterfeit medicines is less than robust, there is broad international consensus that this criminal trade is a serious global public health issue needing immediate action [1,2,4]. Recent investigations and law enforcement efforts have uncovered large-scale illegal counterfeit manufacturing in emerging markets, ties to organized crime and terrorism, and record global seizures in both producing and consuming countries, all associated with patient deaths worldwide [2,4,14,27].

Several key international organizations, including WHO, UNODC, Interpol, and the World Customs Organization (WCO), have attempted to address the global counterfeit medicines issue. Most notably, beginning in 1988, WHO has repeatedly issued resolutions and guidance, while also attempting to be actively engaged in developing policies, programs and governance activities (including its Good Governance for Medicines program) in an attempt to ensure access to safe medicines and combat against counterfeit drugs [3,31]. However, it should be noted that WHO does not have enforcement capabilities, cannot specifically address criminal or law enforcement issues, and generally engages in technical capacity building if resources are available. These limitations hamper any attempted response to the criminal element actively involved in the trade of dangerous counterfeit medicines.

More recently, WHO has struggled to tackle this problem effectively, owing to divergent member state concerns, incompatible ideologies between public health and commercial IPRs, limitations of its current member state-centric governance structures, and lack of adequate resources including challenges from ongoing WHO reform [32,33]. These conflicts have severely hampered a global response to the counterfeit medicines trade, and have necessitated the entry of new international organizations, such as UNODC and Interpol, because of concern of WHO leadership and the need for active, focused, and effective engagement against the criminal networks involved in this trade [34].

Specifically, the attempts by WHO to foster partnerships in combating this illicit trade and engage a broader base of stakeholders has garnered harsh criticism. For example, the WHO-chaired International Medical Products Anti-Counterfeiting Taskforce (IMPACT) brought together a number of governments, international organizations, civil society groups, private sector actors, law enforcement agencies and others to combat this activity in 2006 [35]. However, the future viability of IMPACT is doubtful. Instead, WHA recently adopted the establishment of a new member state mechanism (MSM) in response to criticism regarding perceived associations of WHO with enforcement activities during its relationship with IMPACT, primarily stemming from a customs seizure in the Netherlands of generic pharmaceutical products *en route* from India to Brazil [3,36].

Importantly, MSM is a new governance structure driven exclusively by member state participation, and does not involve active inclusion of important non-member state stakeholders that have been instrumental and active in combating this criminal trade [37]. This regression is symptomatic of larger contextual governance problems within WHO. This includes the failure of the WHA to adopt inclusive governance reforms (specifically, the World Health Forum proposal) to ensure adequate funding and broader stakeholder participation [38]. This reflects the reality that WHO is significantly limited in its ability to engage and leverage resources of multiple stakeholders under its current governance structures, lacks the necessary partnerships to provide crucial information for enforcement operations, and has deficient resources to address the issue programmatically.

### Filling the gap: UNODC, Interpol, and other organizations

In response to WHO governance limitations, other international organizations are filling the gap in institutional capability that WHO is unable to provide. This primarily involves UNODC, which specializes in establishing policy and coordinating actions in combating all forms of transnational organized crime, including crime prevention, criminal justice, corruption, terrorism, and the trade and trafficking of illicit drugs worldwide [39]. All these elements have direct ties to the trade in dangerous counterfeit drugs, and emerging engagement of UNODC has already brought much-needed attention to the fight against this form of transnational pharmaceutical crime. Further, UNODC administers international treaties that can be extended to criminal activities involving dangerous counterfeit medicines, such as the UN Convention against Transnational Organized Crime (UNTOC), which has widespread international adoption, applicability, and viable enforcement mechanisms [3].

Fortunately, UNODC is no stranger to engagement in global health, as evidenced by its mandate to participate in HIV/AIDS prevention through its 'Think AIDS’ campaign and other health activities [40,41]. In addition, Resolution 20/6 by the UN Commission on Crime Prevention and Criminal Justice (CCPCJ) specifically requested and empowered UNODC to engage in the fight against 'fraudulent’ counterfeit medicines by conducting research, providing technical assistance to member states, and co-operation with other international organizations [5]. The CCPCJ resolution in conjunction with a recent UNODC high-level conference of experts on fraudulent medicines makes it clear that UNODC now has an important role to play in global governance responses to dangerous counterfeit medicines [39].

UNODC has also partnered effectively with other international organizations, such as Interpol, WCO, and key actors in the public and private sectors, to lead initiatives that directly target pharmaceutical crime through multi-sectoral law enforcement and border control programs [42]. Importantly, UNODC resolutions have historically supported and gained broad and inclusive stakeholder engagement, extending beyond traditional member state-only inclusion. Avoiding incompatible and divergent member state domestic policy interests by focusing primarily on the transnational criminal aspects of dangerous counterfeit medicines, UNODC represents a more effective forum for stakeholder collaboration and co-operation, but one that has yet to be fully leveraged.

In addition to UNODC activities, Interpol has had a long-standing and effective role in facilitating training, capacity building, investigations, enforcement, and prosecutions against dangerous counterfeit drug purveyors both regionally and globally, and therefore should be sought as an active partner in any response. Interpol has the ability to mobilize law enforcement, customs and border assets, and also engage with both the public and private sectors (including scientific experts, financial institutions, laboratory facilities, and the pharmaceutical industry) to develop intervention packages that support on-the-ground operations aimed at directly disrupting the trade in counterfeit medicines in both the regulated and unregulated global drug supply chain [43,44]. Indeed, Interpol has been the central actor in large global seizures of dangerous drugs, and has recently initiated the creation of the Interpol Pharmaceutical Crime Programme as a comprehensive anti-pharmaceutical crime initiative, in partnership with 29 of the world’s largest pharmaceutical firms [45].

Hence, both UNODC and Interpol have demonstrated an ability to engage effectively with other important non-member state stakeholders on dangerous counterfeit medicines, whereas WHO has experienced challenges. This more inclusive approach has resulted in tangible results, including global co-operation in field operations that have led to counterfeit drug seizures, illicit website closures, arrests, and ongoing investigations [27]. However, despite this relative progression by UNODC and Interpol on the issue, it is also clear that operations and policy formulation need to be informed by evidenced-based data and increased surveillance on the public health and patient safety risks of dangerous counterfeit medicines, which have traditionally been the expertise of organizations such as WHO.

### Enhanced global health governance: UNODC-WHO-Interpol trilateral co-operation mechanism

Currently, the official program activities of UNODC, WHO, and Interpol operate in isolation or through *ad hoc* partnerships that lack a formal and sustained governance mechanism. As an example, neither UNODC nor Interpol have observer status in the new WHO MSM, despite the fact that both of these organizations have institutional experience in cooperating with WHO through initiatives like IMPACT and Interpol operations in the past [34]. This lack of an active co-operation mechanism is worrisome, and provides an indication that WHO may no longer be seeking formal active engagement with other international organizations and actors outside of its MSM, at a time when broader engagement is crucial.

**Figure 1:**
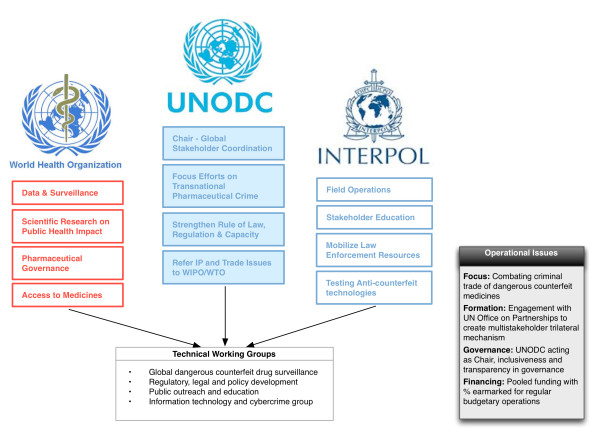
Trilateral working group on counterfeit medicines.

In order to move forward, the respective technical expertise, international legitimacy, financial and non-financial resources, and leveraging of existing partnership networks between these organizations must be coordinated to create a cohesive and transparent governance framework. Given emerging strengths of UNODC as a more inclusive governance forum for broader stakeholder mobilization and its UN mandate through CCPCJ, a trilateral governance mechanism under the auspices of UNODC, with the active participation of WHO and Interpol, should be established [34]. We propose the basic structure and primary roles of the organizations participating in this mechanism in Figure 1.

This could be accomplished by creating a permanent intergovernmental trilateral working group (TWG) composed of UNODC as the chair, and WHO and Interpol as strategic partners. The purpose of the permanent TWG would focus on enabling coordination and co-operation specifically for combating the global trade in dangerous counterfeit medicines, all within the respective mandates and subject matter domains of each organization. A similar governance structure was explored by the WHO working group of member states on SSFFC in September 2011, but was dropped in favor of the current WHO MSM [46].

Fortunately, partnerships within and between UN specialized agencies and other international/intergovernmental organizations are not particularly new. For example, WHO, the World Intellectual Property Organization (WIPO), and the World Trade Organization (WTO) recently engaged in a trilateral co-operation mechanism on the issue of intellectual property and public health, a topic with direct relevance to the global debate on counterfeit medicines [47]. Multiple stakeholder global environmental governance coordinated through UN agencies, such as the United Nations Environmental Programme, have also been used to enhance international co-operation, promote national development planning, provide technical assistance, strengthen laws and institutions, and engage in science-based policy-making for transnational issues regarding the environment [48].

Enabling a pathway for the TWG could be the role of the United Nations Office of Partnerships (UNOP). UNOP serves as a gateway for partnership and alliance opportunities within the UN, as well as engagement with the private industry, foundations, and civil society on issues supporting progress towards achieving the Millennium Development Goals (MDGs) [49]. As the public health threat of counterfeit medicines directly affects population-based health and health-related MDGs as a result of treatment failure, anti-microbial resistance, and patient injury (leading to stunted economic development and productivity), morbidity, and mortality, development of partnership collaborations on dangerous counterfeit medicines would fit into the goals of the programmatic activities of UNOP, and consequentially should be explored.

Specifically, a trilateral UNODC-WHO-Interpol structure would also allow the different organizations to more effectively manage appropriate relationship with their own interest-minded stakeholders, all within their respective domains and internal governance requirements; for example, WHO could manage public health, drug regulatory authority and access to medicine stakeholders; UNODC could interface with private sector actors, customs agencies, and international crime and justice groups; and Interpol could primarily engage with law enforcement officials and engage in public outreach/education campaigns.

Importantly, governance should be transparent, so that the TWG can ensure the legitimacy and appropriate inclusion of necessary stakeholders in its actions. This would allow for specialization of tasks at a high level, and reduce the duplicative efforts that currently operate in parallel under existing initiatives [3]. Further, each organization could establish its own national single points of contact within these subject-area domains. to enable more effective communication and information sharing from the TWG to relevant national authorities for translation of data and global policy development. Resources to fund activities of the TWG could be procured from a variety of sources subject to individual organizational requirements. Hence, WHO could limit participation and funding on issues within its domain that might prove controversial to its member states, whereas UNODC and Interpol might more freely engage in funding mechanisms from both member and non-member states and entities. However, a minimum percentage of all collected funds should be earmarked to support the general operations of the Trilateral WG in order to ensure sustainability and maintain international attention and co-operation against dangerous counterfeit medicines, similar to other proposals for global health governance and financing reform [38].

The TWG could also develop specific technical working groups to address unique and key risk factors in the dangerous counterfeit medicines trade that require the immediate attention and targeted expertise of select participants (Table 1). These groups could include programmatic areas of: 1) global surveillance, pharmacovigilance, and data collection (including development of a central surveillance system, international tracking and tracing systems/standards, and possible development of m-pedigree solutions); 2) regulatory, legal and policy development (to strengthen rule of law, enforcement, and drug regulatory systems); 3) public outreach and education (to inform consumers, patients, healthcare workers, government agencies, and civil society, among others, about the dangers of counterfeit medicine); and 4) information technology and cybercrime (to address specific risk factors such as the link between cybersecurity and crime and illicit Internet-based sourcing). These technical working groups should also examine existing regional or in-country models/tools aimed at combating dangerous counterfeit medicines for potential regional or global scalability.

**Table 1:** Proposed specific technical working groups with the trilateral working group

**Area of focus**	**Description**	**Existing models/tools**	**Goals**
**Global surveillance, pharmacovigilance, and data collection**	Specialized technical group to develop a global, centralized, and harmonized surveillance system for counterfeit medicine detection, reporting, and data sharing. Would also work to develop process for global report on counterfeit medicines	Pharmaceutical Security Institute Counterfeit Incident System. WHO Pilot SSFFC Global Surveillance and Monitoring Project	Establish an active an internationally agreed upon and scientifically validated system for reporting and detection of counterfeit medicines from various sources, including drug regulatory agencies, customs officials, public health agencies, consumers and clinicians, and law enforcement. Would allow for development of data collection from multiple stakeholders that would inform evidence-based policy-making both domestically and globally. Also possibly develop globally harmonized tracking and tracing system, development of e-pedigree/m-pedigree technologies, or best practices from regional or country-level initiatives
**Regulatory, legal and policy development**	Would work to harmonize criminal legal frameworks against counterfeit medicines, harmonize pharmacovigilance activities for counterfeit detection, engage in regulatory capacity building and strengthen pharmaceutical good governance	UNTOC: Application of counterfeit medicines trade as 'serious crime’ under UNTOC existing framework and transnational enforcement tools, Council of Europe MEDICRIME Convention: First International Counterfeit Medicines treaty, UN Environmental Programme Basel Convention on transboundary movement and management of hazardous waste for pharmaceuticals	Aim to develop and implement public policy, laws, and regulations at local, national, regional, and potentially global levels to combat the criminal trade in dangerous counterfeit medicines. Also aim to strengthen pharmaceutical governance to address corruption in health systems associated with drug supply delivery. Should explore existing regulations, laws, legislation, and treaty instruments for possible expansion or immediate application. This specifically includes application of international treaty instruments such as UNTOC, MEDICRIME, and the Basel Convention
**Public outreach and education**	Development of a unified and effective public outreach and education campaign tailored and aimed at a diverse group of stakeholders including consumers/patients, healthcare professionals, law enforcement, drug regulators, customs agents, policymakers, civil society, and other interested parties. Use of multimedia channels and mediums to obtain broadest coverage sensitive to target audience	Interpol Counterfeit Medicines Awareness Campaign 2010, US Food and Drug Administration: BeSafeRx Campaign, National Agency for Food, Drug Administration and Control (Nigeria) educational campaigns, Hong Kong Consumer Council outreach and awareness program on pharmacies establishments detected as selling counterfeit medicines	Fund initiatives to increase global awareness among all interested parties regarding the public health and patient safety dangers of counterfeit medicines. These efforts would attempt to address the demand side of counterfeit medicines from end users, increase engagement on prevention from healthcare professionals, and enable all stakeholders to better report suspect medicines to the relevant authorities
**Information technology and cybercrime**	Special technical working group composed of specialists in information technology, cybersecurity, public health, and law enforcement, with a mandate to specifically address ties between transnational cybercrime and illicit online pharmacies	Interpol Pangea I-VI Multistakeholder Operations.	Development of specific information technology tools to detect and shut down illicit online pharmacies and affiliated third-party enabling technologies. Exploration of existing web monitoring technologies for Internet content surveillance, utilization of tools for technical blocking of violating websites, suspension of financial transactions/processing, and use of other fraud detection tools. Systems that enable verification and certification of legitimate online pharmacies with education of the consumer should also be pursued
		Public-private partnerships: US Alliance for Safe Online Pharmacies; Center for Safe Internet Pharmacies, Integration with current global efforts of the Internet Governance Forum to promote online safety	

The recent emergence of UNODC as an international forum to lead criminal enforcement action against dangerous counterfeit medicines provides an opportunity for better balancing of global health governance through the proposed TWG. Using UNODC as the lead international agency charged with the task of assessing and coordinating stakeholder actions, coalescing specifically around transnational criminal activity of the dangerous counterfeit medicines trade, should also allow WHO the freedom to operate as the expert technical agency it is and redirect global efforts towards crime prevention and patient safety protection.

Importantly, WHO can assess and recommend measures to promote public health issues arising from dangerous counterfeit medicines, and contribute specialized scientific and public health knowledge to this effort. WHO could then concentrate on the core issues of improving access to safe medicines, strengthening health systems for better surveillance, developing monitoring and evaluation protocols for sourcing/distributing safe drugs, strengthening domestic drug regulatory and pharmaceutical governance capacity, and collecting data and studying the epidemiology of counterfeit drugs to enhance the efforts of UNODC and Interpol. Paramount to this effort is the need for more accurate and reliable data on the prevalence of dangerous counterfeit medicines, and surveillance and laboratory capacity for identification. Indeed, proper and robust data collection is fundamental for justifying appropriate regulatory, legal, and law enforcement action. Efforts could include enhancement of data collection under the WHO SSFFC Global Surveillance and Monitoring Project by harmonizing reporting fields and collecting information from a variety of sources (including drug regulatory, customs, and law enforcement authorities) to form a centralized database. This infrastructure would provide a better evidence base for determining the true scope of the global counterfeit medicines trade [50].

This trilateral proposal allows UNODC, in direct partnership with WHO and Interpol, to lead the effort on global enforcement against dangerous counterfeit medicines, independent of contentious IPR considerations, focusing instead on established criminal activities [34]. WHO could objectively identify incidents that present a global public health danger, without consideration of IPRs, and share these findings to this partnership for further assessment based on scientifically validated data. Hence, involvement of WHO would provide crucial public health information to guide criminal enforcement efforts, capacity building, and policy development on the issue, which is largely absent now. UNODC could then actively coordinate and enact policy to address pharmaceutical transnational crime (including potential applicability of UNTOC), with Interpol engaging in educational outreach and mobilizing global law enforcement resources to actively disrupt criminal networks [34]. Trade and IPR disputes would continue to be heard by WTO and WIPO independently within their respective dispute resolution forums. Drugs not fitting the parameters of the dangerous counterfeit drugs group, but that potentially violate IPRs, would be subject to WTO and WIPO dispute resolution procedures and assessed outside of the scope of the public health and criminal activity considerations of the TWG. However, any such assessment should also recognize public health priorities expressed by other international treaties, specifically the WTO Trade-Related Aspects of Intellectual Property Rights (TRIPS) Doha Declaration [51]. These agreements reaffirm rights of member states to exercise TRIPS flexibilities in response to a national health emergency in ensuring equitable access to medicines.

Such division of labor lends itself to better specialization of UN agencies, leveraging their respective institutional strengths and resources, and establishing better global health governance to combat dangerous counterfeit medicines. The union of these specialized UN agencies and intergovernmental organizations under UNODC leadership through the TWG would bring global coverage and enhanced policy coherence to the issue. It would also provide greater legitimacy to actions, given that they are multilateral in nature and not merely regional arrangements. Most importantly, it would separate contentious policy issues such as organized crime and public health from IPR, and trade into appropriate international forums that may act collaboratively towards shared social welfare, equity, and justice goals.

## Conclusion

Recognition, coordination, and active engagement of key stakeholders is essential in combating the global health crisis of dangerous counterfeit medicines. UNODC represents the best forum to engage the multitude of fragmented actors currently addressing this issue. Hence, enhanced global health governance and shared responsibility through a UNODC-WHO-Interpol TWG mechanism may provide a plausible way forward to promote global health security, combat transnational pharmaceutical crime, and, most importantly, ensure safe access to medicines.

## Abbreviations

CCPCJ: UN Commission on Crime Prevention and Criminal Justice; IMPACT: International Medical Products Anti-Counterfeiting Taskforce; Interpol: International Criminal Police Organization; MSM: World Health Organization New Member State Mechanism; OECD: Organisation for Economic Co-operation and Development; PSI: Pharmaceutical Security Institute; SSFFC: Substandard/spurious/falsely-labeled/falsified counterfeit medical products; TRIPS: WTO Trade-Related Aspect of Intellectual Property Rights; UNODC: United Nations Office of Drugs and Crime; UNOP: United Nations Office for Partnerships; UNTOC: United Nations Convention against Transnational Organized Crime; WCO: World Customs Organization; WHA: World Health Assembly; WHO: World Health Organization; WIPO: World Intellectual Property Organization; WTO: World Trade Organization.

## Competing interests

TKM and BAL received journal open access fees support from the Partnership for Safe Medicines (PSM) for this submission, but otherwise received no extramural support from any organization for the submitted work. TKM is the 2011–2013 Carl L. Alsberg MD Fellow of PSM, which supports his general research activities. BAL is a voluntary board member and Vice President of PSM, and receives no compensation for any PSM activities. PSM is not connected with the submitted work. BAL also serves as a member of the National Patient Safety Foundation Research Program Committee, which considers grant proposals addressing medication safety. TKM and BAL report no other relationships or activities that could appear to have influenced the submitted work.

## Author’s contributions

We note that with respect to author contributions, TKM and BAL jointly conceived the study, and jointly wrote and edited the manuscript. Both authors read and approved the final manuscript.

## Pre-publication history

The pre-publication history for this paper can be accessed here:

http://www.biomedcentral.com/1741-7015/11/233/prepub
